# A bivalent mRNA vaccine against RSV infection in rodent models

**DOI:** 10.3389/fimmu.2025.1542592

**Published:** 2025-03-24

**Authors:** Juan Liu, Hanqing Zhao, Wenhao Wang, Binbin Yang, Naifang Zhang, Yu Zhang, Jie Qian, Qiaofang Ma, Yankun Lu, Huafeng Han, Yongsheng Yang

**Affiliations:** Nucleic Acid Medicine Innovation Center, Zhejiang Haichang Biotech Co., Ltd., Hangzhou, Zhejiang, China

**Keywords:** RSV prefusion protein, mRNA vaccines, neutralizing antibody, protection, immunity

## Abstract

Because of the higher conservation of RSV Fusion (F) protein than the glycoprotein (G) across RSV strains and serotypes, the majority of vaccine candidates targets to viral fusion protein (F) rather than glycoprotein to elicit a broader range of protective neutralizing antibodies from infection. In this study, we screened two chemically modified mRNA vaccines expressing RSV prefusion stabilized protein (preF) targeting RSV A2 and B subtypes. After immunization, the antigen-specific binding antibody, neutralizing antibody, and T cell-mediated immune response were evaluated. After challenge with live RSV A2 virus in cotton rats, the protection and safety of vaccine was further evaluated. The results showed that the mRNA vaccine candidates elicited robust antigen-specific binding antibody, neutralizing antibody responses and Th1-biased T-cell responses in both mice and cotton rats. Moreover, cotton rats vaccinated with mRNA vaccine, lung pathology and lung infectious viral loads were significantly reduced, and no vaccine enhanced respiratory disease (VERD) happened. These results collectively demonstrated that mRNA-based vaccine induced strong humoral and cellular immunity, provided outstanding protection against both RSV A2 and RSV B subtypes in rodent animals as well. Our data demonstrated that these mRNA vaccines should be further evaluated in clinical trials.

## Introduction

Respiratory syncytial virus (RSV), as the *Paramyxoviridae* family, is a single nonsegmented negative-stranded RNA virus with a genome encoding 11 different proteins (1–3). RSV is divided into two major antigenic subtypes, RSV A and RSV B, which co-circulate during seasonal epidemics, often with a predominance of one of the subtypes in a single season (4). RSV is associated with upper and lower respiratory tract diseases, and causes high morbidity and mortality among immunocompromised populations, infants, and the elderly worldwide (5). Since 1960, a number of different RSV vaccine have been developed, such as a formalin-inactivated virus vaccine (6), subunit vaccines (3, 7–9), vectored vaccines (2, 4, 10–12), and live attenuated vaccine (13). In addition, some novel vaccine development including F-Fc fusion protein vaccine (14), a nanoparticle vaccine (15), mRNA vaccine (16), and so on, have been in clinical trial in pregnant women. Meanwhile, two licensed monoclonal antibodies targeting the glycoprotein F, Palivizumab and Nirsevimab, can respectively treat high-risk premature-birth infants or healthy infants 8 months of age and younger (17, 18). Currently, three commercial vaccines against RSV in older adults - mRESVIA (Moderna) (19), Abrysvo (Pfizer—BioNTech) (20, 21) and Arexvy (GSK) (22) have been approved for marketing in several countries.

The RSV F protein is a type I fusion glycoprotein and mediates viral entry and contains important neutralizing epitopes (23). The F protein is conserved between the RSV A and RSV B antigenic subgroups of clinical isolates. Thus, Most of the RSV vaccines in clinical trials are based on the F protein (24). During virus-host cell membrane fusion, the F protein transitions from a metastable prefusion conformation to a stable postfusion conformation. RSV postfusion-based vaccines have shown limited or no protective efficacy in clinical trials (25), possibly because the most potent neutralizing antibodies bind to the prefusion conformation (26, 27). However, the development of RSV prefusion-based vaccines has long been hindered due to the instability of the RSV prefusion F protein. To elicit a robust neutralizing antibody response, a few prefusion F proteins were engineered using mutations identified by structure-based design and have demonstrated superior efficacy and immunogenicity in preclinical studies (10, 28, 29).

An internal ribosome entry site (IRES)-mediated system was employed to build polycistronic transcription units in eukaryotic cells that expressed two or more proteins by promoting internal initiation of mRNA translation (30, 31). Previously study reported that up to 4 genes were co-expressed via IRESs (32). In addition, 2A peptides have been used in a very wide range of biotechnological and biomedical applications. During translation, these 2A peptide sequences mediate a eukaryote‐specific, self‐cleaving event with very high efficiency and simultaneously translate a number of proteins at an equal level in all eukaryotic systems (33). We chose an alternative approach for co-expressing multiple genes in one mRNA using IRES or 2A elements.

Messenger RNA (mRNA) technology has certain advantages, and allows the versatile design of vaccine antigens for the rapid generation of candidate antigens for preclinical evaluation, and highly scalable and fast manufacturing as well (34). mRNA vaccines targeting the SARS-CoV-2, Ebola, Zika, and influenza viruses, as well as cancer, have been developed (35).

In our study, we have developed a bivalent mRNA vaccine encoding two stabilized prefusion F proteins of RSV A2 and B and studied the immune mechanisms of protection against RSV infection in rodent models. Firstly, we discovered that mRNA vaccine candidates could induce the production of RSV-specific antibody and generate protective Th1-biased cellular immune response in mice and cotton rats. Secondly, we further illustrated that mRNA vaccine candidates could elicit robust neutralizing antibody responses against RSV A2 and B and reduce viral loads in cotton rats. Additionally, this mRNA vaccine candidate could protect cotton rats against RSV A2 and did not develop vaccine enhanced respiratory disease (VERD). In summary, the data suggest that the mRNA vaccine expressing RSV prefusion F protein could be a safe and effective vaccine candidate for the prevention of RSV infection.

## Materials and methods

### Cells, viruses and animals

Human embryonic kidney 293 cells (HEK293 cells, ATCC CRL-3216) and HEp-2 cells (ATCC CCL-23) were maintained in Dulbecco’s Modified Eagle’s Medium (DMEM, Gibco, 11965092, USA) supplemented with 10% fetal bovine serum (FBS, Gibco, 11965092, USA) and 1% penicillin-streptomycin (Gibco, 15140148, USA). RSV strains A2 and B were obtained from Chongqing Medleader Bio-Pharm Co., Ltd (Chongqing, China) and grown and cultured in HEp-2 cells (ATCC CCL-23). They were used in serum neutralization assays by HEp-2 cells-based plaque assay. RSV strain A2 was also used for challenge study. SPF-grade of 6−8-week-old female BALB/c mice were purchased from Beijing Vital River Laboratory Animal Technology Co., Ltd. Female cotton rats of 7–10 weeks old were purchased from SPF (Beijing) Biotechnology Co., Ltd. and these animals were housed and bred under appropriate temperature and humidity conditions. An animal care and use application for this study was reviewed and approved by Chongqing Bureau of Science and Technology IACUC (SYXK (Yu) 2021-0003).

### mRNA and mRNA-LNP preparations

The recombinant plasmids encoding the T7 RNA polymerase promotor followed by 5′ untranslated region (UTR: AGGAAATAAGAGAGAAAAGAAGAGTAAGAAGAAATATAAGACCCCGGCGCCGCCACC), open reading frame (ORF) containing preF sequences from RSV A2 and RSV B, 3′UTR (GCTGGAGCCTCGGTGGCCTAGCTTCTTGCCCCTTGGGCCTCCCCCCAGCCCCTCCTCCCCTTCCTGCACCCGTACCCCCGTGGTCTTTGAATAAAGTCTGAGTGGGCGGC), and a 110-base poly(A) tail were overexpressed in E. coli, extracted and linearized by restriction endonuclease BspQ I cleavage followed by plasmid recovery and purification. Nucleoside-modified mRNA was synthesized by *in vitro* transcription (IVT). IVT reactions were performed using linearized DNA templates, an optimized T7 RNA polymerase mix (HZYMES biotech, HBP000330, China), ATP (100 mM), GTP (100 mM), CTP (100 mM), N1-Me-pUTP (100 mM), and Cap GAG (m7G (5′) ppp (5′) (2′-OMeA) pG) (Glycogene, CA-1005, China) and purified using an oligo-dT affinity column (Thermo Fisher Scientific, A48352, USA) and Tangential Flow Filtration (TFF, Repligen Corporation, USA). RNA integrity was analyzed by microfluidic capillary electrophoresis (Fragment Analyzer systems 5200, Agilent), and the concentration, pH, and endotoxin level were also determined.

To prepare the mRNA vaccine, the purified mRNA was diluted to the desired concentration with 50 mM citric acid buffer, pH 4.0, to obtain an aqueous solution. LNPs were prepared as described in our previous study (36), and the lipids mixture containing ionizable lipids, 1,2-distearoyl-sn-glycero-3-phosphocholine (DSPC), a polyethylene glycol-lipid and cholesterol were dissolved in ethanol at a molar ratio. Finally, the mRNA vaccine was tested for particle size, polymer dispersity index, mRNA encapsulation, and endotoxin prior to injection into animals. All these parameters were within the acceptable criteria.

### Cell transfection

1×10^6^ HEK293 cells were transfected with T2A-preF and IRES-preF mRNA unit using the Lipofectamine™ Messenger MAX™ transfection reagent (Thermo Fisher Scientific, LMRNA008, USA) in accordance with the manufacturer’s protocol. Briefly, 7.5 µL Lipofectamine™ MessengerMAX™ Reagent was incubated with 125 µL Opti-MEM™ Medium for 10 minutes at room temperature before being mixed with 5 µg of mRNA in 125 µL Opti-MEM™ Medium. After 5 minutes of incubation at room temperature, this mRNA-lipid complex was added to HEK293 cells and incubated for 24 hours at 37°C in 6-well plates. At that point, these cells were harvested and lysed immediately in RIPA buffer (Beyotime, P0013B, China). Cell supernatants were also collected and stored at −20°C until sandwich ELISA analysis.

### Mouse immunizations

Six groups of 10 female BALB/c mice were immunized twice at a 3-week interval with 10 μg DS-Cav1 preF protein (SinoBiological, 11049-VNAS, China) formulated with Adju-phos^®^ (InvivoGen, vac-phos-250, USA) or with the 1 µg or 3 µg or 10 µg dose of candidate mRNA vaccines T2A-preF, IRES-preF, or with saline as a negative control. Two weeks following the second immunization, the serum was collected for serum-specific antibody levels assays. For virus neutralization and ELISpot assays, mice were sacrificed 3 weeks following the second immunization, and the blood and spleens were obtained.

### Sandwich enzyme linked immunosorbent assay (ELISA)

Cell culture supernatant or precipitation lysate or mouse sera were analyzed using the sandwich ELISA kit in accordance with the manufacturer’s instructions (Vazyme, DD3935, China). Briefly, a 96-well ELISA plate (Corning, USA) was coated with anti-RSV pre-F antibody F6-199 in PBS (1 µg/mL, 100 µL/well) at 4°C overnight and incubated with 5% BSA in PBST at 37°C for 1 hour. After four times of wash with PBST, 100 µL thawed cell supernatants or precipitations were added to the wells at 1/5 dilutions in the block buffer. Eight step-wise 2-fold dilutions of the standard samples starting at 0.48 µg/mL concentration were added to the control columns. After washing with PBST, the plate was incubated with horseradish peroxidase (HRP)-conjugated goat anti-mouse IgG (Thermo Fisher Scientific, 31437, USA) at 37°C for 1 hour and developed by adding 100 µL tetramethylbenzidine (TMB) (Invitrogen™, 00-4201-56, USA) for 15 minutes followed by adding 50 µL/well of 2 M sulfuric acid. The absorbance was measured at 450/630 nm on an ELISA microplate reader (TECAN Infinite SPARK, USA).

### Cotton rat immunizations and challenge

Nine groups of five female cotton rats were immunized twice intramuscularly with 4 μg, 10 μg, or 25 μg mRNA vaccine or with an intranasal administration of 1 × 10^6^ pfu RSV A2 or with a formalin-inactivated RSV A2 vaccine (FI-RSV, Taichu Biology) at a 3-week interval and with a naive unvaccinated group. Animals were bled for serology before the immunization and on days 21, 28, and 35. The body weight of the rat was measured every three days before the challenge and on a daily basis after the challenge until humanitarian euthanasia. On day 42 (3 weeks following the second immunization), all animals (excluding 4 μg dose groups) in the study were challenged intranasally with 1 × 106 pfu of RSV A2 strain delivered in a 0.1 mL volume. On day 47, all animals were euthanized and lung tissues were isolated and trisected to process for virus quantification of viral load, pathology, and cytokine mRNA analysis. The cotton rat VERD study was conducted at Chongqing Medleader Bio-Pharm Co., Ltd (Chongqing, China). The naive unvaccinated group was challenged to serve as a control for the pathological changes associated with natural infection.

### ELISA

The level of anti-RSV preF antibody in the vaccinated serum was determined essentially with an ELISA method as described in the reference (16). Briefly, Ninety-six-well ELISA plates (Corning, USA) were coated with 2 μg/mL purified preF recombinant protein and incubated at 4°C overnight. The plates were then washed and blocked for 1 hour at room temperature with 5% non-fat milk dissolved in PBST. Sera from mice were serially diluted 2-fold starting at 1:12800 (1:12800, 1:25600, 1:51200, 1:102400, 1:204800, 1:409600, 1:819200, 1:1638400, 1:3276800) in the blocking buffer, or sera from cotton rats were serially diluted 3-fold starting at 1:300 (1:300, 1:900, 1:2700, 1:8100, 1:24300, 1:72900, 1:218700, 1:656100, 1:1968300) in the blocking buffer, transferred to each well and incubated for 2 hours at room temperature. Following the plate wash, the plates were incubated with HRP-conjugated rabbit anti-mouse (Thermo Fisher Scientific, PA1-28568, USA) or chicken anti-rat IgG (Thermo Fisher Scientific, A18727, USA) antibody, diluted at 1:100 in the block buffer for 1 hour at room temperature. The plates were developed with TMB and the reaction was stopped within 10 min and the absorbance was read at 450 nm on an ELISA microplate reader. The optical density (OD) value of the highest dilution was 2.1 times higher than that of the negative control at the same dilution and was employed as the serum endpoint dilution titer. Each experiment was conducted three times.

### Serum neutralization assays

The serum neutralization assay of live RSV strains A2 and B on HEp-2 cells was determined by 50% plaque reduction neutralization titers (PRNT_50_) in both BALB/c mice and cotton rats. Similar protocols were delineated in reference (37). Briefly, sera were heat-inactivated and serially diluted in a 96-well plate. The sera were mixed in a 1:1 ratio with RSV stocks and incubated for 1 hour at 37°C. The virus-serum mixtures were then added to HEp-2 cells in 96-well plates, incubated for 1 hour, and subsequently overlaid with carboxymethyl cellulose and incubated for 5 days at 37°C and 5% CO2. Cells were then washed, fixed, permeabilized, and blocked. Each well was then incubated with anti-mouse or cotton rat RSV preF protein monoclonal antibody at 37°C for 1 hour, followed by HRP-conjugated rabbit anti-mouse or chicken anti-rat IgG antibody. The cells were washed and developed with TMB. Plaques were counted with an AID ELISpot Reader Classic and analyzed by the ELISpot 7.0 iSpot software (AID, Germany). PRNT_50_ was defined as the highest reciprocal serum dilution that inhibited more than 50% of the plaques observed in infected control wells without serum treatment on the same plate (the mean of four control wells per plate was used).

### Enzyme-Linked ImmunoSPOT (ELISpot) assay

Mice or cotton rats were euthanized, and their spleens were removed under aseptic conditions. To detect specific T lymphocyte responses, the IFN-γ/IL-4 ELISpot assay was carried out using the IFN-γ/IL-4 ELISpot-plus Kit in accordance with the manufacturer’s instructions (Dakewe, 2210004 for Mouse IFN-γ Precoated ELISpot Kit, 2210402 for Mouse IL-4 Precoated ELISpot Kit, China; Mabtech, 3220–4APW-10 for the Rat IFN-γ ELISpot-plus Kit, Sweden). 2 × 10^5^ splenocytes and RSV A2 full-length preF peptide mix (0.5 μg/mL, GenScript, China) were added to 96-well ELISpot plates precoated with IFN-γ/IL-4 antibodies. After incubation at 37°C for 20 hours, the plates were washed with wash buffer, and biotinylated anti-mouse or rat IFN-γ/IL-4 antibody was added to each well at 37°C for 1 hour, followed by the addition of streptavidin-HRP and incubation at 37°C for 1 hour. After the addition of chromogenic substrate for 5 minutes at room temperature, the reaction was halted with pure water for 2 minutes, and the plates were dried for 30 minutes at room temperature. Finally, the results of the ELISpot assays were evaluated using an AID ELISpot Reader Classic and analyzed by the ELISpot 7.0 iSpot software (AID, Germany).

### Viral load

Lung samples were determined by plaque assay on HEp-2 cells. Briefly, samples were diluted and added into 96-well plates as described (38). The diluted samples were added into 96-well plates with confluent HEp-2 cells monolayers for 1 hour at 37°C, and carboxymethyl cellulose was also added. After 3 days at 37°C, cells were further processed as described above. Finally, viral plaques were counted with an ELISPOT reader and analyzed using the AID ELISpot Reader Classic and analyzed by the ELISpot 7.0 iSpot software (AID, Germany), and virus titers were expressed as pfu/g of tissue.

### Hematoxylin and eosin (H&E) staining

The right lobe of the lung tissues was dissected and inflated with 10% neutral buffered formalin to its normal volume and fixed in the same fixative solution for 24 hours, and was cut into 4 µm tissue sections and stained with H&E for histopathological examination. The slides were evaluated for evidence of peribronchiolitis (inflammatory cell infiltration around the bronchioles), perivasculitis (inflammatory cell infiltration around the small blood vessels), interstitial pneumonia (inflammatory cell infiltration and thickening of alveolar walls), and alveolitis (cells within the alveolar spaces). Slides were scored on a scale of 0 (normal) to 4 (severe). Images were captured using a Pannoramic^®^ 250 Flash III (3D HISTECH, Hungary) and rendered using CaseViewer V2.4.0.

### QRT-PCR

The relative level of cytokine mRNA was determined via quantitative real-time PCR (qRT-PCR). Briefly, the middle lobe of the right lung tissue was homogenized, and total RNA was extracted using the TRIZOL Reagent (Pufei Biotech, 3101–100, China) in accordance with the manufacturer’s instructions. One μg of total RNA was used to synthesize cDNA using the RevertAid First Strand cDNA Synthesis Kit (Thermo Fisher Scientific, K1621, USA). The qPCR reaction was performed using the ChamQ™ Universal SYBR^®^ qPCR Master Mix (Vazyme, Q711–02, China) and a LightCycler96 real-time PCR system (Roche, Basel, Switzerland). The reaction was performed as follows: 95°C for 300 seconds; 40 cycles of 95°C for 10 seconds and 60°C for 30 seconds; 95°C for 10 seconds, 65°C for 60 seconds and 97°C for 1 second; 37°C for 30 seconds. The relative expression units were subsequently normalized to the level of glyceraldehyde-3-phosphatedehydrogenase (GAPDH) mRNA expressed in the corresponding sample. Quantitative analysis was conducted using the LightCycler96 software with a relative quantification method (ΔΔCt) to assess the level of cytokine mRNA.

### Bronchoalveolar lavage fluid (BALF) analysis

BALF was collected, stained, and counted using methods previously delineated (3, 39). Briefly, BALF was collected from cotton rats immediately after CO_2_ asphyxiation by washing the lung with 2 mL of sterile saline. The lavaged sample from each cotton rat was kept on ice until needed. BALF was centrifuged at 300 × g for 5 minutes, and cells were separated. After centrifugation, pellets were re-suspended in 1 mL PBS, and centrifuged at 300 × g for 5 minutes again. Pellets were re-suspended in 0.3 mL PBS. BALF cell counts were determined by hemocytometer, and BALF differentials were determined on Diff-Quik (Solarbio, G1541, China). Differential cell counting was performed using standard morphological criteria. By using flow cytometry, the relative size and granularity for the BALF cell were analyzed based on the side-scattering and forward-scattering.

### Statistical analysis

Statistically significant differences between groups and treatments were ascertained by two-way analysis of variance (ANOVA), and those between groups were determined by one-way ANOVA with the Tukey multiple comparison test and using GraphPad Prism 8 software (GraphPad Software, Inc., La Jolla, CA, USA). A *p* value of less than 0.05 was regarded as statistically significant.

## Results

### mRNA antigen design and expression

**Figure 1:**
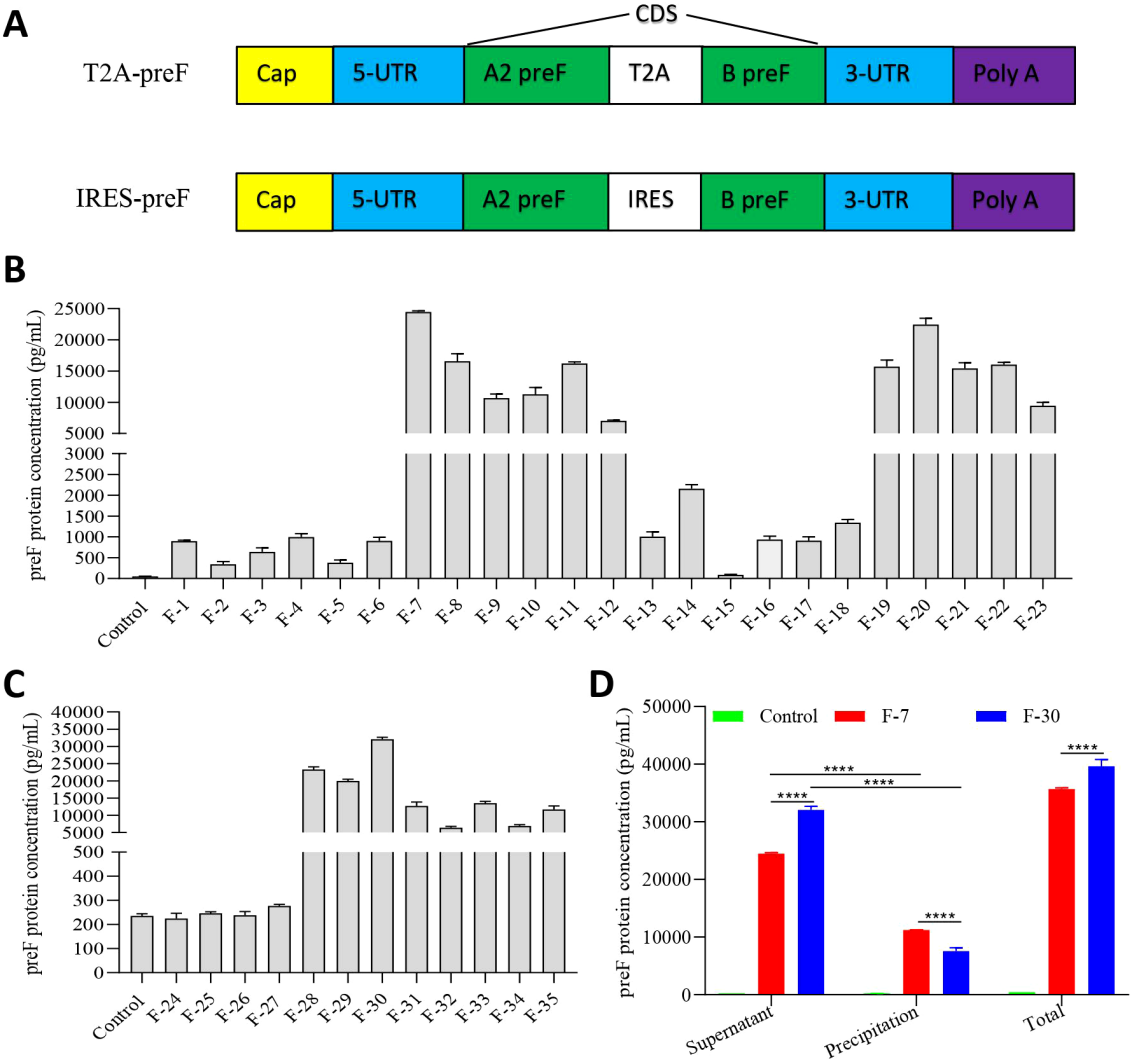
The design of mRNA antigen and expression. **(A)** Structure of RSV mRNA Vaccine. UTR: untranslated region; preF: prefusion F protein; the coding domain sequence is composed of A2 preF and B preF and connected by T2A or IRES. **(B-D)** The expression of mRNA constructs in HEK293 cells was analyzed by sandwich ELISA (*n* = 5). The data are presented as the mean ± standard error of the mean (SEM). All the data are representative of three independent experiments. A one-way **(B, C)** or two-way **(D)** ANOVA with Tukey’s multiple comparisons test was conducted, ****p < 0.0001.

The RSV fusion protein is the principal target for virus-neutralizing antibodies, and most of the neutralizing antibodies are directed against the preF conformation following natural infection in humans (27). Therefore, preF becomes the antigen targeted by the majority of RSV vaccine candidates. In our study, to improve its expression and stabilize the desired conformation as well, we designed 35 mRNA antigen sequences based on preF. Among them, 23 sequences (F-1~F-23) were designed on T2A-preF, and 12 sequences (F-24~F-35) were designed on IRES-preF. Briefly, the mRNA construct encompassed a 5′ cap structure, a 5′ UTR, one open reading frame (ORF) including the A2 preF and B preF sequence connected by the T2A or IRES sequence, a 3′ UTR, and a poly(A) tail. The ORF in this study encoded the prefusion protein of the RSV A2 strain (GenBank: KT992094) and RSV B strain (GenBank: AF013254) (**Figure 1A**). Sequence optimization on these 35 mRNA constructs was necessary for cellular preF protein expression. All parameters of these mRNA qualities were within the acceptable criteria. We then compared the antigen expression induced by these optimized mRNA constructs in cell transfection. Sandwich ELISA analysis disclosed that the expression of the constructs F-7 or F-30 was the highest in the cell culture supernatant among these constructs (**Figures 1B, C**). To further investigate the expression of the constructs F-7 or F-30, we explored the preF expression in cell precipitation and culture supernatant by the same method. The results revealed that the expression of the preF protein induced by F-7 mRNA in cell precipitation was 1.5-fold higher than that induced by F-30 mRNA. However, compared with F-30 mRNA, the expression of the preF protein in the cell culture supernatant induced by F-7 mRNA decreased by 1.3 folds. Of note, the expression of the total preF in the F-7 mRNA group was 1.1-fold lower than that in the F-30 mRNA group, and the expression of the preF protein induced by F-7 or F-30 mRNA in the cell culture supernatant was 2.1 or 4.2-fold higher than that in cell precipitation, respectively (**Figure 1D**). In addition, we selected 11 constructs with high antigen expression from the 23 constructs of T2A-preF and conducted a mouse vaccination experiment. After serum collection at 24 hours, the antigen expression of preF *in vivo* was detected. Consistent with the *in vitro* data, the *in vivo* results also indicated that only the construct F7 was the group with the optimal antigen expression (**Supplementary Figure S1**). We designated the two constructs, F-7 and F-30, as T2A-preF and IRES-preF mRNA, respectively. In conclusion, the mRNA sequences with high expression that we screened from 35 sequences lay a foundation for further research on the immunogenicity of the mRNA vaccine.

### mRNA vaccines elicit robust humoral and cellular immune responses in mice

**Figure 2:**
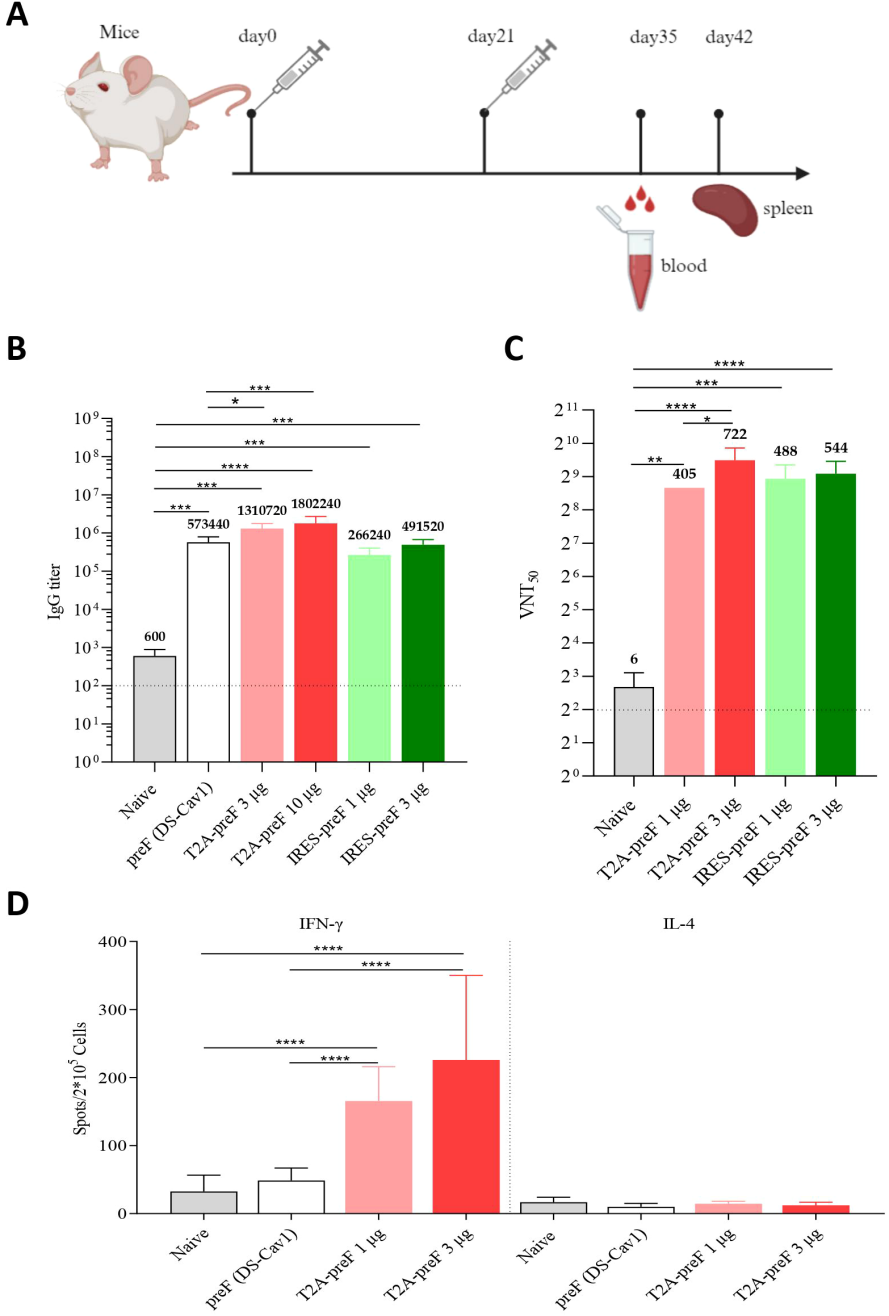
Mouse immunogenicity. **(A)** Immunization Schedule. Groups of 6- to 8-week-old female naive BALB/c mice (*n* = 10) were vaccinated via intramuscular injection with the indicated doses of RSV mRNA vaccine at 3-week intervals. Blood collection and spleen extraction were performed at the predefined time points after immunization. **(B)** The preF protein-specific IgG levels were quantified by ELISA (*n* = 5). **(C)** Neutralization assays of the live RSV A2 were quantified (*n* = 5). **(D)** Protein-specific T cell ELISpot assay results. Splenocytes were stimulated with overlapping peptide pools spanning the RSV A2 full-length F protein at a final concentration of 0.5 μg/mL (*n* = 10). The data are presented as the mean ± SEM. The horizontal dashed line indicates the lower limit of quantification. All the data are representative of three independent experiments. A one-way **(B, C)** or two-way **(D)** ANOVA with Tukey’s multiple comparisons test was conducted, *p < 0.05; **p < 0.01; ***p < 0.001; ****p < 0.0001.

To evaluate the immunogenicity of the RSV mRNA vaccine in mice, BALB/c mice were intramuscularly immunized twice with T2A-preF (1 µg, 3 µg, or 10 µg) or IRES-preF (1 µg or 3 µg) mRNA vaccine at 3-week intervals. Immunization with 10 μg of the DS-Cav1 protein (preF) antigen formulated with an aluminum phosphate-based adjuvant was added as a positive control, and the naive group (saline, nonvaccination group) was served as a negative control. Sera were collected at the indicated time points and tested for antigen-specific IgG levels by ELISA and the viral neutralization antibody against RSV A2 by serum neutralization assay (**Figure 2A**). The results demonstrated that the mRNA vaccine induced high levels of RSV F protein-specific IgG and serum neutralizing antibody titers compared to the control. For the T2A-preF mRNA vaccine, the serological immune responses were 2.3–4.1-fold higher than vaccinations using the aluminum adjuvanted DS-Cav1 protein (**Figures 2B, C**).

Three weeks after the boost immunization with the T2A-preF mRNA vaccine, the mice were sacrificed, and the splenocytes were harvested and analyzed by enzyme-linked immunospot assay (ELISpot). As shown in **Figure 2D**, ELISpot results showed that the IFN-γ secretion by T lymphocytes from spleens was significantly higher in the 1 µg or 3 µg groups compared to the control. Notably, no differences in IL-4 secretion by T lymphocytes from spleens were observed in all immunized mice compared to the control (**Figure 2D**). In summary, the RSV mRNA vaccine successfully induces both humoral and Th1-biased cellular immune responses in mice, making it an ideal candidate for RSV mRNA vaccine development.

### mRNA vaccines elicit strong humoral and cellular immune responses in cotton rats

**Figure 3:**
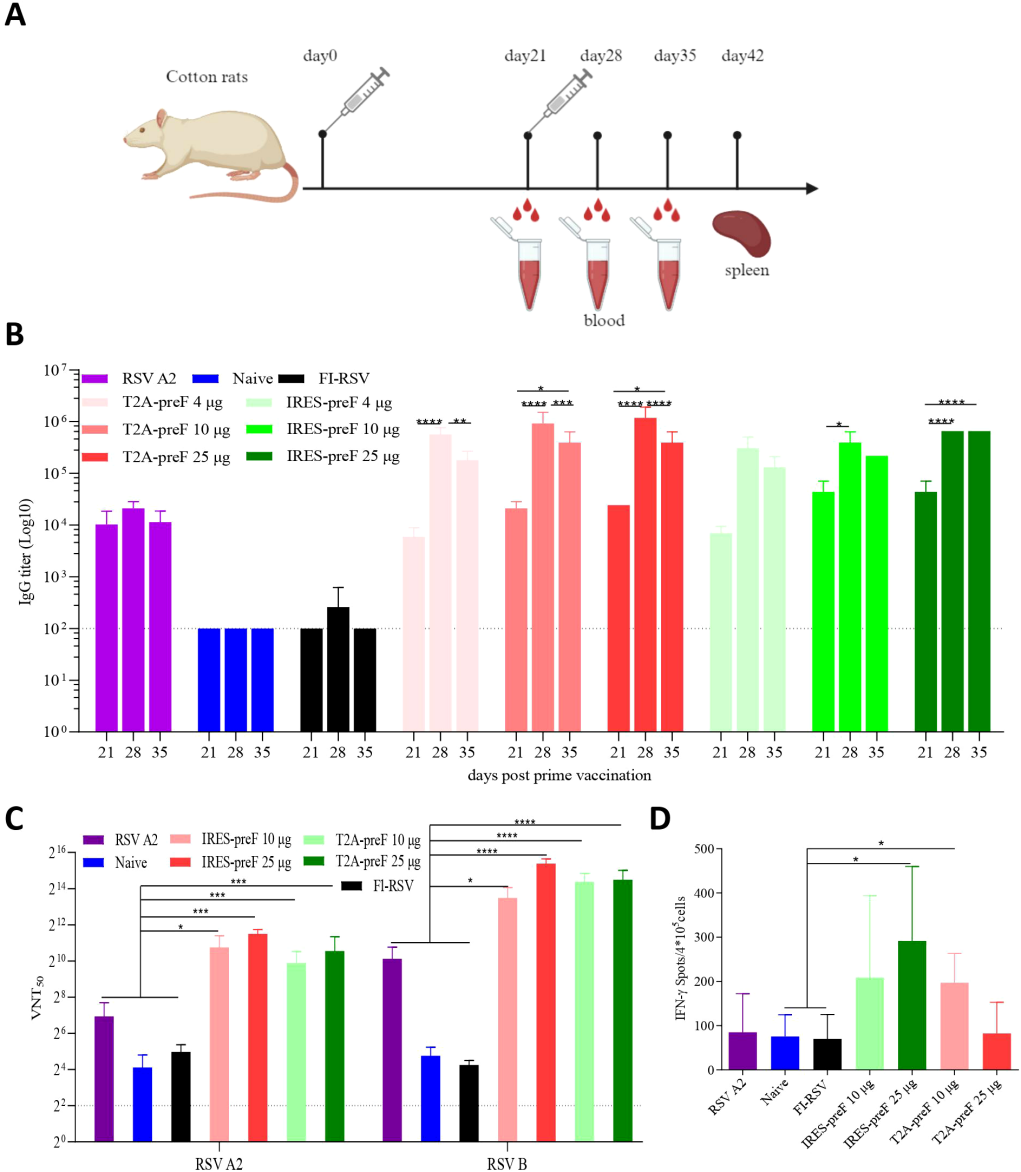
Cotton rat immunogenicity. **(A)** Immunization Schedule. Groups of 7- to 10-week-old female naive Cotton rats (*n* = 5) were vaccinated via intramuscular injection with the indicated doses of the RSV mRNA vaccine at 3-week intervals. Blood collection and spleen extraction were performed at the predefined time points after immunization. **(B)** The preF protein-specific IgG levels were quantified by ELISA (*n* = 5). **(C)** Neutralization assays of the live RSV A2 and RSV B virus were quantified (*n* = 5). **(D)** Protein-specific T cell ELISpot assay results. Splenocytes were stimulated with overlapping peptide pools spanning the RSV A2 full-length F protein protein at a final concentration of 0.5 μg/mL (*n* = 5). The data are presented as the mean ± SEM. The horizontal dashed line indicates the lower limit of quantification. All data are representative of three independent experiments. A one-way **(D)** or two-way **(B, C)** ANOVA with Tukey’s multiple comparisons test was conducted, *p < 0.05; **p < 0.01; ***p < 0.001; ****p < 0.0001.

To further explore vaccine immunogenicity in cotton rats, the T2A-preF or IRES-preF mRNA vaccine (4 µg, 10 µg, or 25 µg) or FI-RSV were intramuscularly vaccinated twice to cotton rats 3 weeks apart. Groups of cotton rats were intranasally immunized once with RSV A2 as a positive control and with PBS as a negative control. Sera were collected at various times following the second immunization and tested for the binding antibody titer against the prefusion F protein by ELISA, and for the neutralization of RSV A2 or RSV B (**Figure 3A**). The results indicated that all mRNA vaccines elicited elevated levels of serum antibodies to RSV F proteins along with serum neutralizing antibody titers. Irrespective of the dose, the antibody titer induced by the T2A-preF mRNA vaccine reached a peak at day 28 and declined significantly at day 35, yet remained higher than those at day 21, and the same trend was witnessed in the IRES-preF mRNA vaccine group (**Figure 3B**, **Supplementary Figure S2**).

The mRNA antigens utilized in this study were designed based on RSV A2 and RSV B sequences. Although the F protein is conserved between RSV A2 and RSV B genotypes, the neutralizing antibody titers against the two genotypes should be demonstrated. All of the mRNA vaccines elicited significantly higher neutralizing antibody titers against RSV A2 or RSV B strain compared with FI-RSV, the naive, or RSV A2 groups. Nevertheless, there was no dose dependency (**Figure 3C**). Additionally, ELISpot results indicated that the IFN-γ secretion by T lymphocytes from spleens was significantly higher in the 10 µg T2A-preF or 25 µg IRES-preF mRNA groups compared to the naive or FI-RSV groups, which was consistent with the observation in mice (**Figure 3D**). Collectively, these results demonstrated that the RSV mRNA vaccine induced a strong humoral and Th1-biased cellular immune response in cotton rats.

### mRNA vaccines protect cotton rats against RSV A2 challenge

**Figure 4:**
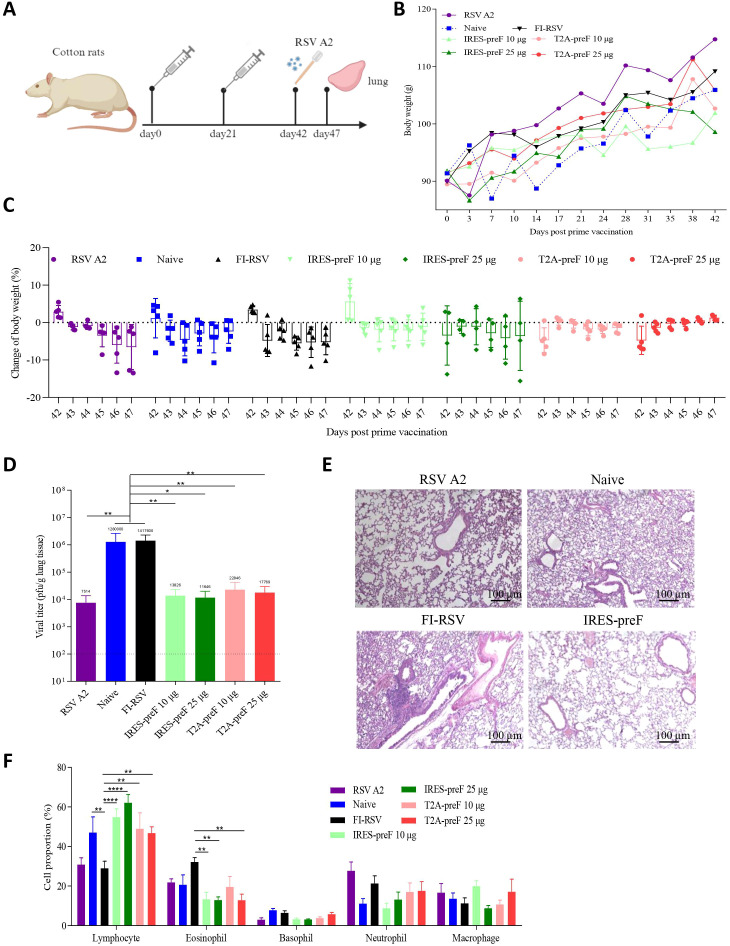
Evaluation of the immune protection provided by RSV mRNA vaccine during *in vivo* challenge. **(A)** Immunization and Challenge Schedule. 7- to 10-week-old female cotton rats were immunized with two doses of the vaccines via the intramuscular route at 3-week intervals (*n* = 5). Subsequently, all animals were challenged with live RSV A2 at 42 days post-vaccination, and the lung tissues were collected at day 47 after immunization. **(B, C)** The body weights of the cotton rats were monitored and documented before and after the challenge (*n* = 5). The mice were euthanized after observation. **(D)** Viral loads in the lung tissues of challenged rats were measured with qRT-PCR at day 47 (*n* = 5). **(E)** H&E staining was performed to assess pathological changes in the lungs of cotton rats (*n* = 5). **(F)** The proportion of lymphocytes, eosinophils, basophils, neutrophils and macrophages in the BALF was analyzed by Diff-Quik assay (*n* = 5). The data are presented as the mean ± SEM. The horizontal dashed line indicates the lower limit of quantification. All the data are representative of three independent experiments. A one-way **(D)** or two-way **(B, C, F)** ANOVA with Tukey’s multiple comparisons test was conducted, *p < 0.05; **p < 0.01; ****p < 0.0001.

To assess the ability of candidate vaccines to provide protection against RSV A2 challenge, cotton rats immunized with T2A-preF or IRES-preF mRNA vaccine were challenged with RSV A2 following two immunizations, in contrast to RSV A2 infected, naive unimmunized, or FI-RSV immunized animals (**Figure 4A**). The body weight of cotton rats was measured every 3 days, and the results showed that the body weight generally increased over time. Meanwhile, the body weight change after the challenge was also tested, and the results showed that the rats in all the mRNA vaccine groups except the IRES-preF mRNA vaccine group showed varying degrees of body weight increase one day after the challenge, suggesting that the mRNA vaccines were safe to a certain extent (**Figures 4B, C**). Besides the body weight, we also evaluated the virus load in cotton rat lungs on day 5 after the challenge. The viral loads in the mRNA vaccine groups were significantly reduced compared with that in the FI-RSV immunized group (**Figure 4D**).

To further observe the pathological changes in the lungs after RSV A2 challenge, we selected the animals immunized with 25 µg IRES-preF mRNA vaccine for H&E staining and the animals with RSV A2 infected, naive unimmunized, or FI-RSV immunized as controls. Consistently, no significant pathological changes in the lungs of cotton rats were observed in the IRES-preF mRNA vaccine group, while alveolar damage, inflammatory cellular infiltration, and hemorrhage were observed in the alveolar space of animals immunized with these controls (**Figure 4E**). In addition to histological inflammation, we also examined the proportion of inflammatory cells in the airways of BALF 5 days after RSV A2 challenge. The Diff-Quik assay results showed that mRNA vaccine stimulation significantly increased the proportion of lymphocytes in the BALF compared to the FI-RSV group. While administration of the mRNA vaccine significantly reduced the eosinophils proportion, no significant differences were observed on basophils, macrophages, and neutrophils cell proportions (**Figure 4F**). Likewise, we also used flow cytometry to find that the proportion of only lymphocytes was enhanced and granulocytes was decreased in the mRNA vaccine groups compared with the FI-RSV group (**Supplementary Figure S3**). Therefore, these results suggested that mRNA vaccine immunization does not cause pulmonary histopathology and eosinophilia upon RSV challenge.

### mRNA vaccine immunization does not cause VERD and upregulation of Th2 cytokines in cotton rat

**Figure 5:**
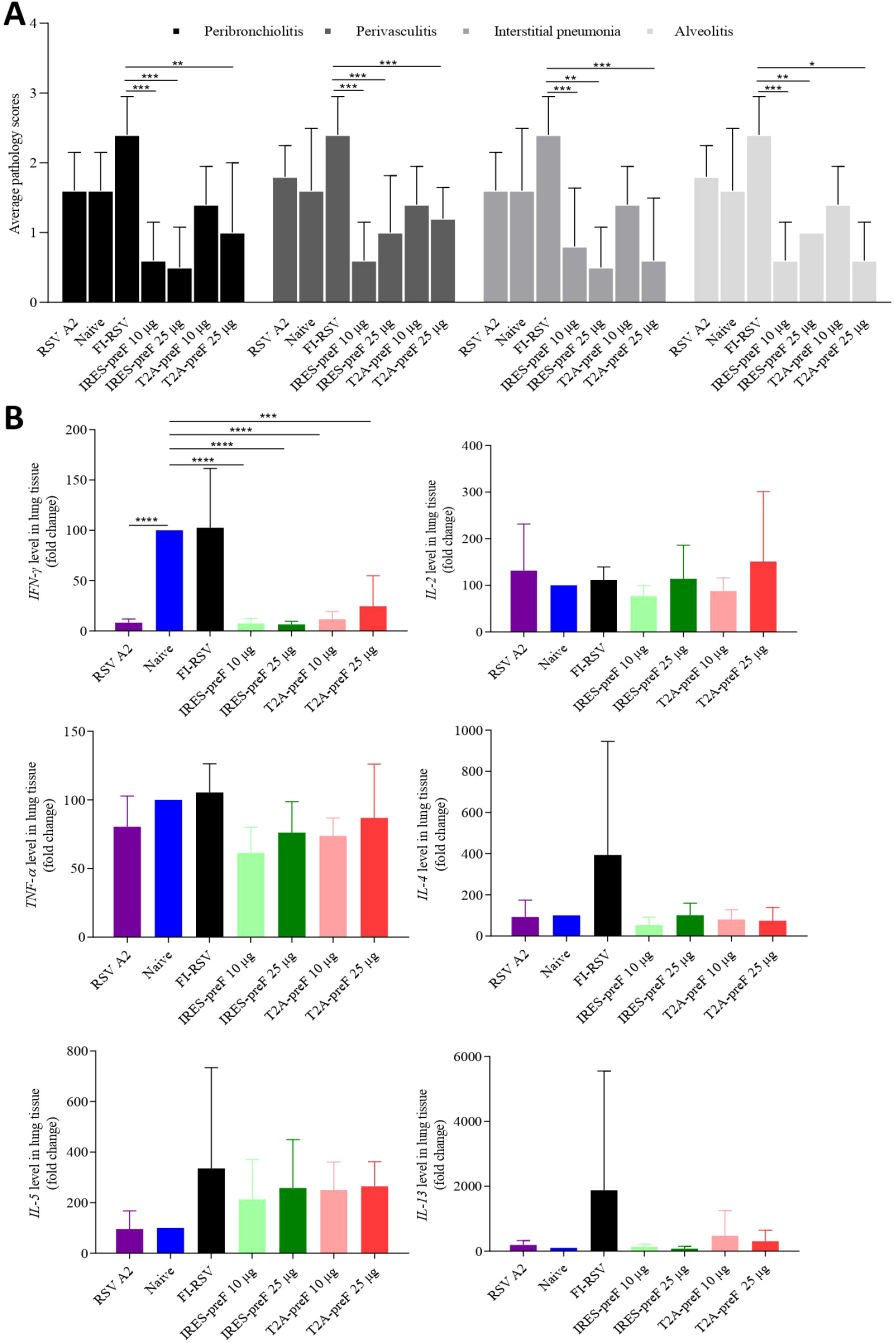
Histopathological changes of cotton rat lungs after RSV challenge. **(A)** Lung tissue sections stained with H&E were scored for peribronchiolitis, perivasculitis, interstitial pneumonia, and alveolitis from 0 (no pathology) to 4 (severe pathology) in accordance with diagnostic criteria (*n* = 5). **(B)** The expression of cytokine genes IFN-γ, IL-2, TNF-α, IL-4, IL-5, and IL-13 in the lung tissue was determined by qRT-PCR (*n* = 5). The data are presented as the mean ± SEM. All the data are representative of three independent experiments. A one-way **(B)** or two-way **(A)** ANOVA with Tukey’s multiple comparisons test was conducted, *p < 0.05; **p < 0.01; ***p < 0.001; ****p < 0.0001.

To determine pulmonary histopathology, lung tissue was evaluated for evidence of peribronchiolitis, perivasculitis, interstitial pneumonia, or alveolitis as well as the mean pathology scores on a scale of 0–4 according to diagnostic criteria (**Figure 5A**). Cotton rats vaccinated with the FI-RSV vaccine showed the highest pathology scores among all four pathologies, and animals vaccinated with T2A-preF mRNA or with IRES-preF mRNA vaccine showed lower severity score levels compared with the FI-RSV vaccine. However, no obvious differences in pathology scores were observed between the naive group and the other four mRNA vaccine immunized groups.

Previous studies have reported that VERD is attributed to the biased Th2 type of CD4 cells induced by FI-RSV vaccination in mouse models (39, 40), so the cytokine expression in lung tissue obtained from cotton rats was evaluated in the study. The mRNA from the lung of the cotton rat was extracted and evaluated for the expression of Th1 (IFN-γ, TNF-α, IL-2) and Th2 (IL-4, IL-5, and IL-13) -associated cytokines. As expected, the expression of mRNA for IFN-γ, TNF-α, IL-2, IL-4, IL-5, and IL-13 was the highest in the group vaccinated with the FI-RSV vaccine, while only IFN-γ mRNA expression in animals immunized with T2A-preF or IRES-preF mRNA vaccine was significantly lower compared to the naive group (**Figure 5B**). These results indicated that mRNA vaccine vaccination was safe and did not lead to the upregulation of Th2 cytokines following the challenge.

## Discussion

RSV is a major cause of severe respiratory disease in infants and the elderly globally. A safe and effective RSV vaccine is a major unmet medical need. Here, we have evaluated mRNA vaccines expressing two constructs of RSV F in mouse and cotton rat models. Two-dose immunization of T2A-preF or IRES-preF mRNA vaccine was capable of inducing the neutralizing antibodies against the RSV A2 or B strain in mice or cotton rats. ELISpot analysis demonstrated that T2A-preF or IRES-preF mRNA vaccine could induce RSV-specific T cell immunities and Th1 activation in mice and cotton rats. T2A-preF or IRES-preF mRNA vaccine could significantly reduce the viral loads of RSV A2 in the lung tissue and prevent the lung histopathology induced by RSV A2 infection in cotton rats. Finally, the mRNA vaccine did not lead to VERD in cotton rats. Based on these data, it can be concluded that the T2A-preF or IRES-preF mRNA vaccine is capable of eliciting significant humoral and cellular immune responses against RSV.

The use of IRES and/or the 2A peptide to generate such multicistronic vectors has become the norm for tagging endogenous genes with reporter or recombinase proteins (41–43). Hence, we designed two mRNA vaccine candidates that were fused with RSV A2 preF and RSV B preF linked by IRES or T2A to assess their expression. The results revealed a significant difference between T2A-preF and IRES-preF constructs and a lower expression level of the IRES-preF construct in cell precipitation. One possible explanation was the lower efficiency of IRES-driven translation caused by the considerable length of the natural IRES. However, the expression of T2A-preF in the supernatant is lower than that of IRES-preF, and one reason could be mainly due to incomplete cleavage of the 2A peptide, which is consistent with the previous study (44).

Current research indicates that RSV mRNA vaccine induces both cellular and humoral immunities (16). Antibody production is also one of the most important components of the adaptive immune response. An adjuvanted recombinant RSV-F subunit vaccine induced higher or comparable IgG antibody (~10^6^) and neutralizing antibody levels (~2^8^) (45). An RSV A-based monovalent Ad26/preF protein vaccine induced IgG antibody (~10^4^) and neutralizing antibodies (~2^15^), as well as protection against both RSV A and RSV B subtypes in animals (4). mRNA vaccine candidates expressing either prefusion stabilized or native forms of RSV F protein elicited robust IgG antibody (~10^7^) and neutralizing antibody (~10^4^) responses in both mice and cotton rats. Another mRNA vaccine, mRNA-1345 from Moderna, also elicited RSV neutralizing antibody titers (~10^4^) (46). In our study, a novel bivalent mRNA vaccine could induce a high level of IgG antibody (~10^6^) and neutralizing antibody (~2^9^) in mice, and a comparable level of IgG antibody (~10^6^) and neutralizing antibody (~2^15^) in cotton rats. In comparison with other studies, our study demonstrated antibody titers that were marginally lower than those of Moderna but significantly higher than those of other vaccines. Of note, in terms of determining neutralizing antibody titer, we observed that the levels of neutralizing antibodies against subtype B were higher than those against subtype A2. This difference may be attributed to variations in the viral dosage used for neutralizing antibody assessment. Besides antibody, a Th1-biased cellular immune response is preferred for the development of the RSV vaccine (24, 47). As for the cellular immune response, a robust CD4^+^ and CD8^+^ T cell response to RSV F peptides was observed in all mice immunized with mRNA expressing forms of RSV F (16). In our study, the ELISpot assay demonstrated that the T2A-preF mRNA vaccine could induce RSV-specific T cell response and Th1 activation in mice or cotton rats, despite the absence of IL-4 in cotton rats due to antibody indiscriminability.

Safety for the development of RSV vaccines is another major consideration. Early research indicated that FI-RSV has led to VERD mainly due to low neutralizing antibody responses and Th2-biased T cell responses (48). Cotton rats are more prone to RSV infection than mice throughout their lifespan and have been widely utilized to assess candidate vaccines for efficacy and safety before clinical testing (49–51). In our studies, cotton rats immunized with FI-RSV exhibited the most severe lung inflammation in all disease parameters, including lung pathology scores around the peribronchiolitis, perivasculitis, interstitial pneumonia, and alveolitis. Additionally, FI-RSV vaccination induced the highest proportion of eosinophil cells among these leukocytes after RSV challenge. Live RSV A2 -infected cotton rats presented histopathology similar to the naive group with RSV infection. Cotton rats vaccinated with T2A-preF or IRES-preF mRNA vaccine had lung pathology scores, but was not significantly different from unimmunized animals after RSV challenge. More importantly, these scores were significantly lower than those from cotton rats immunized with FI-RSV, which was consistent with the previous study (3, 16).

In addition, we evaluated cytokine gene expression in the lungs of the vaccinated or unvaccinated animals after RSV challenge. IFN-γ-producing T cells were shown to contribute to RSV protection as well as disease, and high levels of IFN-γ might contribute to RSV vaccine-enhanced disease and inflammation (52). We observed that IFN-γ was upregulated in naive unvaccinated animals and those vaccinated with FI-RSV following RSV challenge, and the expression of the Th2-associated cytokines IL-4, IL-5, and IL-13 was upregulated in animals vaccinated with FI-RSV, but not in naive unvaccinated controls. In contrast, only IFN-γ was expressed in the lung of cotton rats vaccinated with T2A-preF or IRES-preF mRNA vaccine after RSV challenge, which was significantly reduced compared with naive unvaccinated animals. The findings in this study suggest that both mRNA vaccines are able to protect cotton rats from RSV infection and prevent the accompanying inflammatory response associated with infection as well.

Currently, there are some potential risks associated with age-specific or long-term safe RSV vaccines in clinical trials, particularly rare adverse reactions that may occur over the course of long-term use (53–56). Although no obvious safety problems were observed in our animal experiments, its safety for long-term use needs further study and monitoring.

In conclusion, our results collectively demonstrate that our mRNA vaccines expressing RSV preF protein could induce neutralizing antibodies and provide protection against both RSV A2 and RSV B subtypes. Further research will assess the protective mechanism of mRNA vaccines, including the functional subsets of CD4^+^ T cells and tissue resident memory T cells in the lungs before and after the challenge, and the evaluation of the ability of these vaccine candidates to enhance the immune response to RSV in previously exposed animals as a model for adult vaccination. Our findings provide valuable information regarding mRNA vaccines in the design and clinical transformation. This implies that these vaccine candidates can confer protective clinical efficacy against both RSV subtypes.

## Data Availability

The raw data supporting the conclusions of this article will be made available by the authors, without undue reservation.

## Supplementary material

Supplementary Figure 1 The RSV preF protein expression of mRNA constructs *in vivo*. Mice were intramuscularly immunized with different mRNA vaccines based on T2A-preF (3 μg and 10 μg), and 24 hours later, the sera were collected for antigen expression analysis by sandwich ELISA (*n* = 5). The data are presented as the mean ± standard error of the mean (SEM). All the data are representative of three independent experiments.

Supplementary Figure 2 The preF protein-specific IgG level was quantified by ELISA (*n* = 5). The data are presented as the mean ± SEM. The horizontal dashed line indicates the lower limit of quantification. All the data are representative of three independent experiments. A two-way ANOVA with Tukey’s multiple comparisons test was conducted, **p* < 0.05; ***p* < 0.01; ****p* < 0.001; *****p* < 0.0001.

Supplementary Figure 3 The proportion of lymphocytes, granulocytes and macrophages in the BALF was analyzed by flow cytometry (*n* = 5). The data are presented as the mean ± SEM. All the data are representative of three independent experiments. A two-way ANOVA with Tukey’s multiple comparisons test was conducted, **p* < 0.05; ***p* < 0.01.

## References

[1] Hall CB Long CE Schnabel KC. Respiratory syncytial virus infections in previously healthy working adults. Clin Infect Dis. (2001) 33:792–6. doi: 10.1086/322657 11512084

[2] Grieves JL Yin Z Garcia-Sastre A Mena I Peeples ME Risman HP. A viral-vectored RSV vaccine induces long-lived humoral immunity in cotton rats. Vaccine. (2018) 36:3842–52. doi: 10.1016/j.vaccine.2018.04.089 PMC5990485 29779923

[3] Hwang HS Lee YT Kim KH Park S Kwon YM Lee Y. Combined virus-like particle and fusion protein-encoding DNA vaccination of cotton rats induces protection against respiratory syncytial virus without causing vaccine-enhanced disease. Virology. (2016) 494:215–24. doi: 10.1016/j.virol.2016.04.014 27123586

[4] Cox F Saeland E Thoma A van den Hoogen W Tettero L Drijver J. RSV A2-based prefusion F vaccine candidates induce RSV A and RSV B cross binding and neutralizing antibodies and provide protection against RSV A and RSV B challenge in preclinical models. Vaccines (Basel). (2023) 11(3):672. doi: 10.3390/vaccines11030672 36992257 PMC10057437

[5] Gonzalez PA Bueno SM Carreno LJ Riedel CA Kalergis AM. Respiratory syncytial virus infection and immunity. Rev Med Virol. (2012) 22:230–44. doi: 10.1002/rmv.1704 22290692

[6] Chin J Magoffin RL Shearer LA Schieble JH Lennette EH. Field evaluation of a respiratory syncytial virus vaccine and a trivalent parainfluenza virus vaccine in a pediatric population. Am J Epidemiol. (1969) 89:449–63. doi: 10.1093/oxfordjournals.aje.a120957 4305200

[7] Zhao B Yang J He B Li X Yan H Liu S. A safe and effective mucosal RSV vaccine in mice consisting of RSV phosphoprotein and flagellin variant. Cell Rep. (2021) 36:109401. doi: 10.1016/j.celrep.2021.109401 34289371

[8] Bouzya B Rouxel RN Sacconnay L Mascolo R Nols L Quique S. Immunogenicity of an AS01-adjuvanted respiratory syncytial virus prefusion F (RSVPreF3) vaccine in animal models. NPJ Vaccines. (2023) 8:143. doi: 10.1038/s41541-023-00729-4 37773185 PMC10541443

[9] McGinnes Cullen L Luo B Wen Z Zhang L Durr E Morrison TG. The respiratory syncytial virus (RSV) G protein enhances the immune responses to the RSV F protein in an enveloped virus-like particle vaccine candidate. J Virol. (2023) 97:e0190022. doi: 10.1128/jvi.01900-22 36602367 PMC9888267

[10] van der Fits L Bolder R Heemskerk-van-der-Meer M Drijver J van Polanen Y Serroyen J. Adenovector 26 encoded prefusion conformation stabilized RSV-F protein induces long-lasting Th1-biased immunity in neonatal mice. NPJ Vaccines. (2020) 5:49. doi: 10.1038/s41541-020-0200-y 32566260 PMC7293210

[11] Salisch NC Izquierdo Gil A Czapska-Casey DN Vorthoren L Serroyen J Tolboom J. Adenovectors encoding RSV-F protein induce durable and mucosal immunity in macaques after two intramuscular administrations. NPJ Vaccines. (2019) 4:54. doi: 10.1038/s41541-019-0150-4 31885877 PMC6925274

[12] Saeland E van der Fits L Bolder R Heemskerk-van-der-Meer M Drijver J van Polanen Y. Combination Ad26.RSV.preF/preF protein vaccine induces superior protective immunity compared with individual vaccine components in preclinical models. NPJ Vaccines. (2023) 8:45. doi: 10.1038/s41541-023-00637-7 36949051 PMC10033289

[13] Endt K Wollmann Y Haug J Bernig C Feigl M Heiseke A. A recombinant MVA-based RSV vaccine induces T-cell and antibody responses that cooperate in the protection against RSV infection. Front Immunol. (2022) 13:841471. doi: 10.3389/fimmu.2022.841471 35774800 PMC9238321

[14] Zhang Y Zhou Z Zhu SL Zu X Wang Z Zhang LK. A novel RSV F-Fc fusion protein vaccine reduces lung injury induced by respiratory syncytial virus infection. Antiviral Res. (2019) 165:11–22. doi: 10.1016/j.antiviral.2019.02.017 30822450

[15] Bergeron HC Murray J Juarez MG Nangle SJ DuBois RM Tripp RA. Immunogenicity and protective efficacy of an RSV G S177Q central conserved domain nanoparticle vaccine. Front Immunol. (2023) 14:1215323. doi: 10.3389/fimmu.2023.1215323 37457705 PMC10338877

[16] Espeseth AS Cejas PJ Citron MP Wang D DiStefano DJ Callahan C. Modified mRNA/lipid nanoparticle-based vaccines expressing respiratory syncytial virus F protein variants are immunogenic and protective in rodent models of RSV infection. NPJ Vaccines. (2020) 5:16. doi: 10.1038/s41541-020-0163-z 32128257 PMC7021756

[17] Griffin MP Yuan Y Takas T Domachowske JB Madhi SA Manzoni P. Single-dose nirsevimab for prevention of RSV in preterm infants. N Engl J Med. (2020) 383:415–25. doi: 10.1056/NEJMoa1913556 32726528

[18] Meissner HC Welliver RC Chartrand SA Law BJ Weisman LE Dorkin HL. Immunoprophylaxis with palivizumab, a humanized respiratory syncytial virus monoclonal antibody, for prevention of respiratory syncytial virus infection in high risk infants: a consensus opinion. Pediatr Infect Dis J. (1999) 18:223–31. doi: 10.1097/00006454-199903000-00004 10093942

[19] Goswami J Baqui AH Doreski PA Perez Marc G Jimenez G Ahmed S. Humoral immunogenicity of mRNA-1345 RSV vaccine in older adults. J Infect Dis. (2024) 230(5):e996–e1006. doi: 10.1093/infdis/jiae316 PMC11566230 38889247

[20] Nodelman M Scott AM. RSVpreF (Abrysvo) and nirsevimab-alip (Beyfortus) for the prevention of respiratory syncytial virus infection. Am Fam Physician. (2024) 109:578–9. 38905561

[21] Shaukat A Batool UEA Nasser N. A new era in maternal-child health: Abrysvo’s role in RSV prevention. Health Sci Rep. (2024) 7:e2236. doi: 10.1002/hsr2.2236 38966073 PMC11222291

[22] Abbasi HQ Oduoye MO. Revitalizing hope for older adults: The use of the novel Arexvy for immunization against respiratory syncytial virus. Health Sci Rep. (2023) 6:e1648. doi: 10.1002/hsr2.1648 37916140 PMC10617982

[23] Graham BS Modjarrad K McLellan JS. Novel antigens for RSV vaccines. Curr Opin Immunol. (2015) 35:30–8. doi: 10.1016/j.coi.2015.04.005 PMC4553118 26070108

[24] Mazur NI Higgins D Nunes MC Melero JA Langedijk AC Horsley N. The respiratory syncytial virus vaccine landscape: lessons from the graveyard and promising candidates. Lancet Infect Dis. (2018) 18:e295–311. doi: 10.1016/S1473-3099(18)30292-5 29914800

[25] Falloon J Yu J Esser MT Villafana T Yu L Dubovsky F. An adjuvanted, postfusion F protein-based vaccine did not prevent respiratory syncytial virus illness in older adults. J Infect Dis. (2017) 216:1362–70. doi: 10.1093/infdis/jix503 PMC5853767 29029260

[26] Magro M Mas V Chappell K Vazquez M Cano O Luque D. Neutralizing antibodies against the preactive form of respiratory syncytial virus fusion protein offer unique possibilities for clinical intervention. Proc Natl Acad Sci U S A. (2012) 109:3089–94. doi: 10.1073/pnas.1115941109 PMC3286924 22323598

[27] Ngwuta JO Chen M Modjarrad K Joyce MG Kanekiyo M Kumar A. Prefusion F-specific antibodies determine the magnitude of RSV neutralizing activity in human sera. Sci Transl Med. (2015) 7:309ra162. doi: 10.1126/scitranslmed.aac4241 PMC4672383 26468324

[28] Krarup A Truan D Furmanova-Hollenstein P Bogaert L Bouchier P Bisschop IJM. A highly stable prefusion RSV F vaccine derived from structural analysis of the fusion mechanism. Nat Commun. (2015) 6:8143. doi: 10.1038/ncomms9143 PMC4569726 26333350

[29] Steff AM Monroe J Friedrich K Chandramouli S Nguyen TL Tian S. Pre-fusion RSV F strongly boosts pre-fusion specific neutralizing responses in cattle pre-exposed to bovine RSV. Nat Commun. (2017) 8:1085. doi: 10.1038/s41467-017-01092-4 29057917 PMC5651886

[30] Shaimardanova AA Kitaeva KV Abdrakhmanova II Chernov VM Rutland CS Rizvanov AA. Production and application of multicistronic constructs for various human disease therapies. Pharmaceutics. (2019) 11(11):580. doi: 10.3390/pharmaceutics11110580 31698727 PMC6920891

[31] Douin V Bornes S Creancier L Rochaix P Favre G Prats AC. Use and comparison of different internal ribosomal entry sites (IRES) in tricistronic retroviral vectors. BMC Biotechnol. (2004) 4:16. doi: 10.1186/1472-6750-4-16 15279677 PMC514710

[32] Fussenegger M. The impact of mammalian gene regulation concepts on functional genomic research, metabolic engineering, and advanced gene therapies. Biotechnol Prog. (2001) 17:1–51. doi: 10.1021/bp000129c 11170478

[33] Luke GA Ryan MD. Therapeutic applications of the ‘NPGP’ family of viral 2As. Rev Med Virol. (2018) 28:e2001. doi: 10.1002/rmv.2001 30094875

[34] Vogel AB Kanevsky I Che Y Swanson KA Muik A Vormehr M. BNT162b vaccines protect rhesus macaques from SARS-CoV-2. Nature. (2021) 592:283–9. doi: 10.1038/s41586-021-03275-y 33524990

[35] Sun W He L Zhang H Tian X Bai Z Sun L. The self-assembled nanoparticle-based trimeric RBD mRNA vaccine elicits robust and durable protective immunity against SARS-CoV-2 in mice. Signal Transduct Target Ther. (2021) 6:340. doi: 10.1038/s41392-021-00750-w 34504054 PMC8426336

[36] Liu J Han H Yang B Zhang N Li J Chen X. Immunogenicity and protective efficacy of the HC009 mRNA vaccine against SARS-CoV-2. Front Immunol. (2024) 15:1416375. doi: 10.3389/fimmu.2024.1416375 39131158 PMC11310568

[37] GeurtsvanKessel CH Okba NMA Igloi Z Bogers S Embregts CWE Laksono BM. An evaluation of COVID-19 serological assays informs future diagnostics and exposure assessment. Nat Commun. (2020) 11:3436. doi: 10.1038/s41467-020-17317-y 32632160 PMC7338506

[38] Citron MP Patel M Purcell M Lin SA Rubins DJ McQuade P. A novel method for strict intranasal delivery of non-replicating RSV vaccines in cotton rats and non-human primates. Vaccine. (2018) 36:2876–85. doi: 10.1016/j.vaccine.2018.02.110 29599087

[39] Delgado MF Coviello S Monsalvo AC Melendi GA Hernandez JZ Batalle JP. Lack of antibody affinity maturation due to poor Toll-like receptor stimulation leads to enhanced respiratory syncytial virus disease. Nat Med. (2009) 15:34–41. doi: 10.1038/nm.1894 19079256 PMC2987729

[40] Graham BS Henderson GS Tang YW Lu X Neuzil KM Colley DG. Priming immunization determines T helper cytokine mRNA expression patterns in lungs of mice challenged with respiratory syncytial virus. J Immunol. (1993) 151:2032–40. doi: 10.4049/jimmunol.151.4.2032 8345194

[41] Ghim CM Lee SK Takayama S Mitchell RJ. The art of reporter proteins in science: past, present and future applications. BMB Rep. (2010) 43:451–60. doi: 10.5483/bmbrep.2010.43.7.451 20663405

[42] Provost E Rhee J Leach SD. Viral 2A peptides allow expression of multiple proteins from a single ORF in transgenic zebrafish embryos. Genesis. (2007) 45:625–9. doi: 10.1002/dvg.20338 17941043

[43] Furler S Paterna JC Weibel M Bueler H. Recombinant AAV vectors containing the foot and mouth disease virus 2A sequence confer efficient bicistronic gene expression in cultured cells and rat substantia nigra neurons. Gene Ther. (2001) 8:864–73. doi: 10.1038/sj.gt.3301469 11423934

[44] Ho SC Bardor M Li B Lee JJ Song Z Tong YW. Comparison of internal ribosome entry site (IRES) and Furin-2A (F2A) for monoclonal antibody expression level and quality in CHO cells. PloS One. (2013) 8:e63247. doi: 10.1371/journal.pone.0063247 23704898 PMC3660568

[45] Bian L Zheng Y Guo X Li D Zhou J Jing L. Intramuscular inoculation of AS02-adjuvanted respiratory syncytial virus (RSV) F subunit vaccine shows better efficiency and safety than subcutaneous inoculation in BALB/c mice. Front Immunol. (2022) 13:938598. doi: 10.3389/fimmu.2022.938598 35935960 PMC9354885

[46] Moderna. Serum neutralizing titer in BALB/c mice using mRNA-1345 and mRNA-1777 (2020). Available online at: https://www.sec.gov/Archives/edgar/data/1682852/000168285221000006/mrna-20201231.htm accessed (December 31, 2020).

[47] Soto JA Stephens LM Waldstein KA Canedo-Marroquin G Varga SM Kalergis AM. Current insights in the development of efficacious vaccines against RSV. Front Immunol. (2020) 11:1507. doi: 10.3389/fimmu.2020.01507 32765520 PMC7379152

[48] Polack FP Teng MN Collins PL Prince GA Exner M Regele H. A role for immune complexes in enhanced respiratory syncytial virus disease. J Exp Med. (2002) 196:859–65. doi: 10.1084/jem.20020781 PMC2194058 12235218

[49] Johnson S Oliver C Prince GA Hemming VG Pfarr DS Wang SC. Development of a humanized monoclonal antibody (MEDI-493) with potent *in vitro* and *in vivo* activity against respiratory syncytial virus. J Infect Dis. (1997) 176:1215–24. doi: 10.1086/514115 9359721

[50] Prince GA Jenson AB Hemming VG Murphy BR Walsh EE Horswood RL. Enhancement of respiratory syncytial virus pulmonary pathology in cotton rats by prior intramuscular inoculation of formalin-inactiva ted virus. J Virol. (1986) 57:721–8. doi: 10.1128/JVI.57.3.721-728.1986 PMC252798 2419587

[51] Siber GR Leombruno D Leszczynski J McIver J Bodkin D Gonin R. Comparison of antibody concentrations and protective activity of respiratory syncytial virus immune globulin and conventional immune globulin. J Infect Dis. (1994) 169:1368–73. doi: 10.1093/infdis/169.6.1368 8195619

[52] Castilow EM Olson MR Meyerholz DK Varga SM. Differential role of gamma interferon in inhibiting pulmonary eosinophilia and exacerbating systemic disease in fusion protein-immunized mice undergoing challenge infection with respiratory syncytial virus. J Virol. (2008) 82:2196–207. doi: 10.1128/JVI.01949-07 PMC2258940 18094193

[53] Ison MG Papi A Athan E Feldman RG Langley JM Lee DG. Efficacy and safety of respiratory syncytial virus (RSV) prefusion F protein vaccine (RSVPreF3 OA) in older adults over 2 RSV seasons. Clin Infect Dis. (2024) 78:1732–44. doi: 10.1093/cid/ciae010 PMC11175669 38253338

[54] Wilson E Goswami J Baqui AH Doreski PA Perez-Marc G Zaman K. Efficacy and safety of an mRNA-based RSV preF vaccine in older adults. N Engl J Med. (2023) 389:2233–44. doi: 10.1056/NEJMoa2307079 38091530

[55] Kampmann B Madhi SA Munjal I Simoes EAF Pahud BA Llapur C. Bivalent prefusion F vaccine in pregnancy to prevent RSV illness in infants. N Engl J Med. (2023) 388:1451–64. doi: 10.1056/NEJMoa2216480 37018474

[56] Walsh EE Perez Marc G Zareba AM Falsey AR Jiang Q Patton M. Efficacy and safety of a bivalent RSV prefusion F vaccine in older adults. N Engl J Med. (2023) 388:1465–77. doi: 10.1056/NEJMoa2213836 37018468

