# Supplementary figures and images for: A bivalent mRNA vaccine against RSV infection in rodent models
Source: Front Immunol. 2025 Mar 24;16:1542592. doi: 10.3389/fimmu.2025.1542592 (PMC11974254; doi:10.3389/fimmu.2025.1542592)

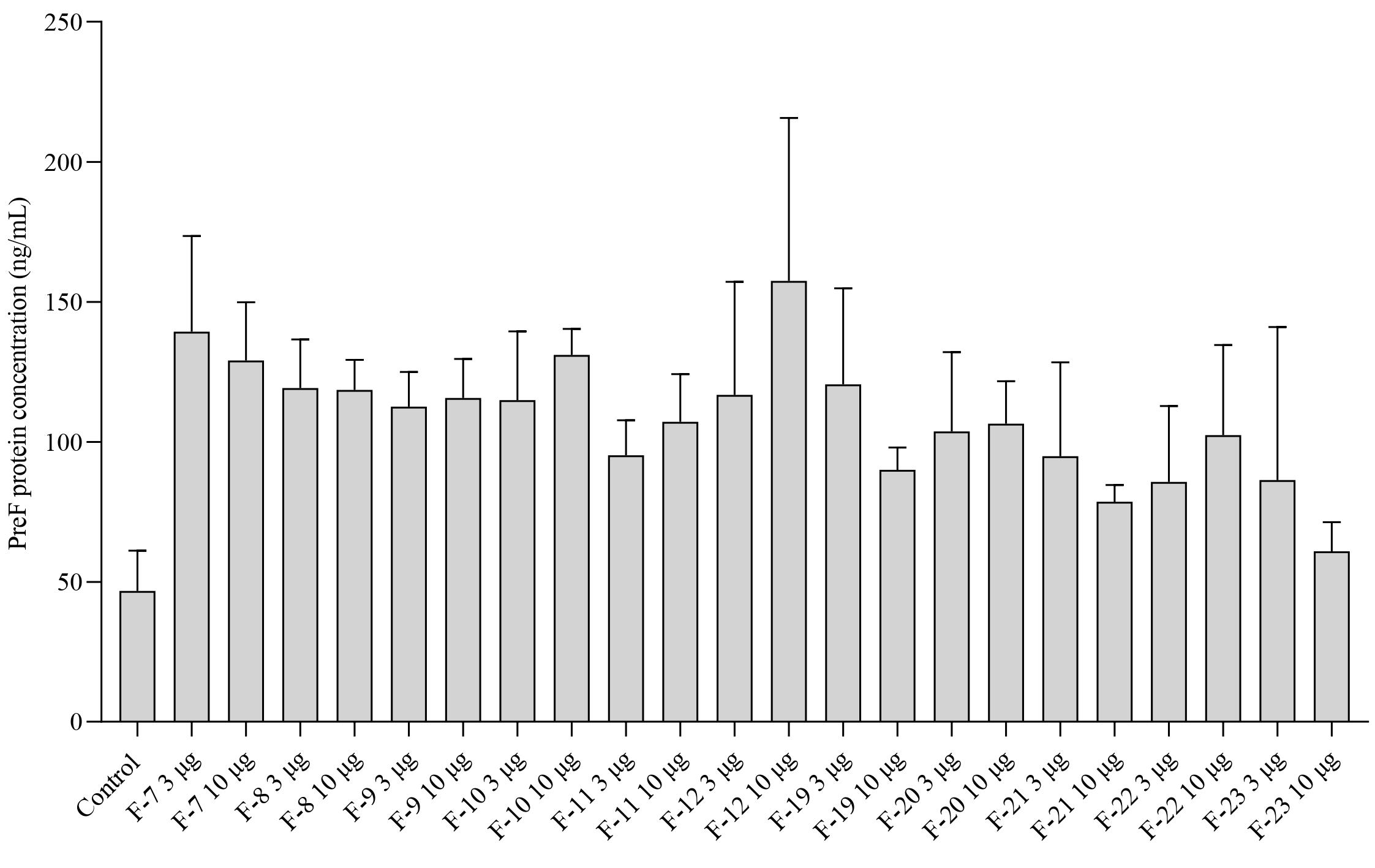

Supplement: Supplementary Figure 1 — The RSV preF protein expression of mRNA constructs in vivo. Mice were intramuscularly immunized with different mRNA vaccines based on T2A-preF (3 μg and 10 μg), and 24 hours later, the sera were collected for antigen expression analysis by sandwich ELISA (n = 5). The data are presented as the mean ± standard error of the mean (SEM). All the data are representative of three independent experiments. [file Image1.jpeg]

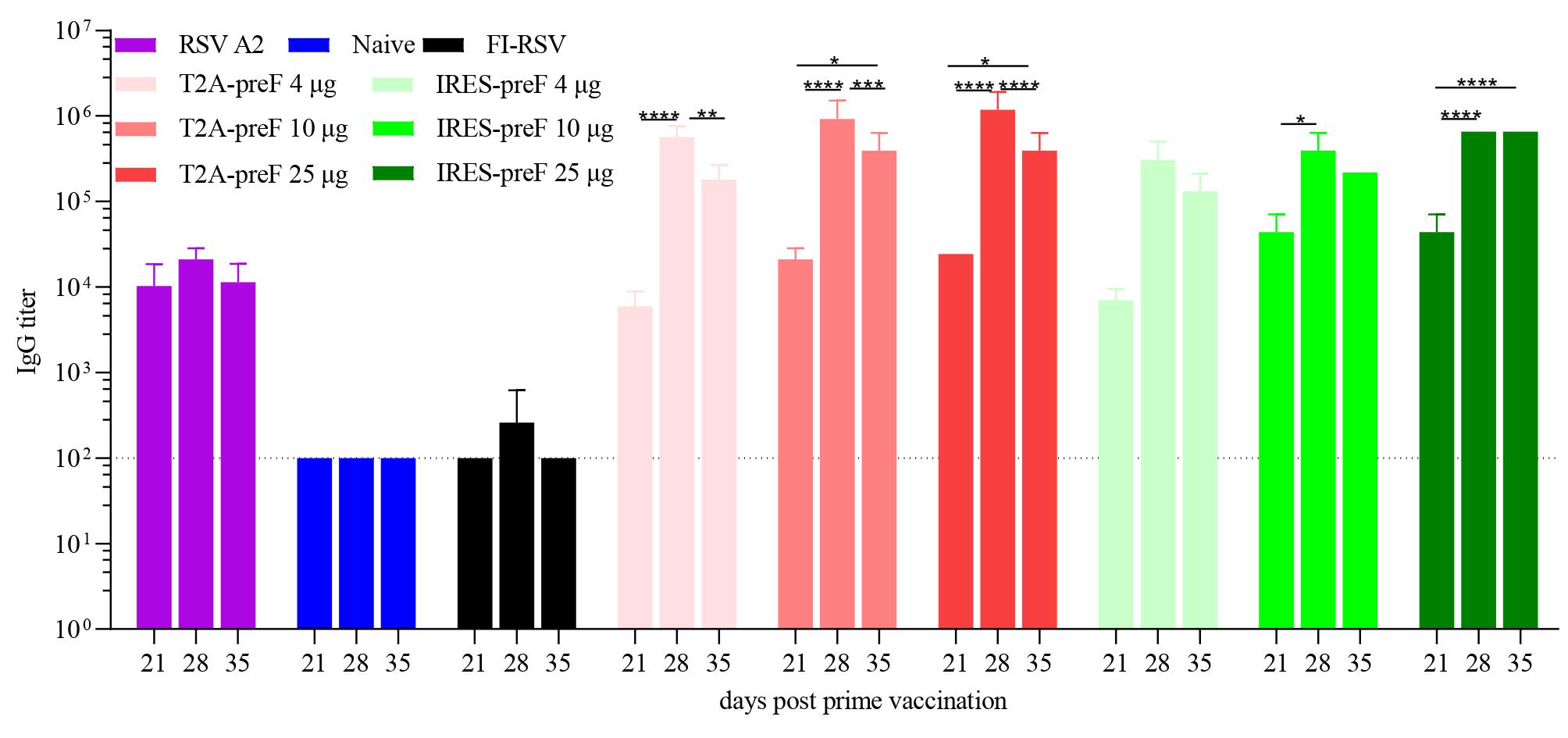

Supplement: Supplementary Figure 2 — The preF protein-specific IgG level was quantified by ELISA (n = 5). The data are presented as the mean ± SEM. The horizontal dashed line indicates the lower limit of quantification. All the data are representative of three independent experiments. A two-way ANOVA with Tukey’s multiple comparisons test was conducted, *p < 0.05; **p < 0.01; ***p < 0.001; ****p < 0.0001. [file Image2.jpeg]

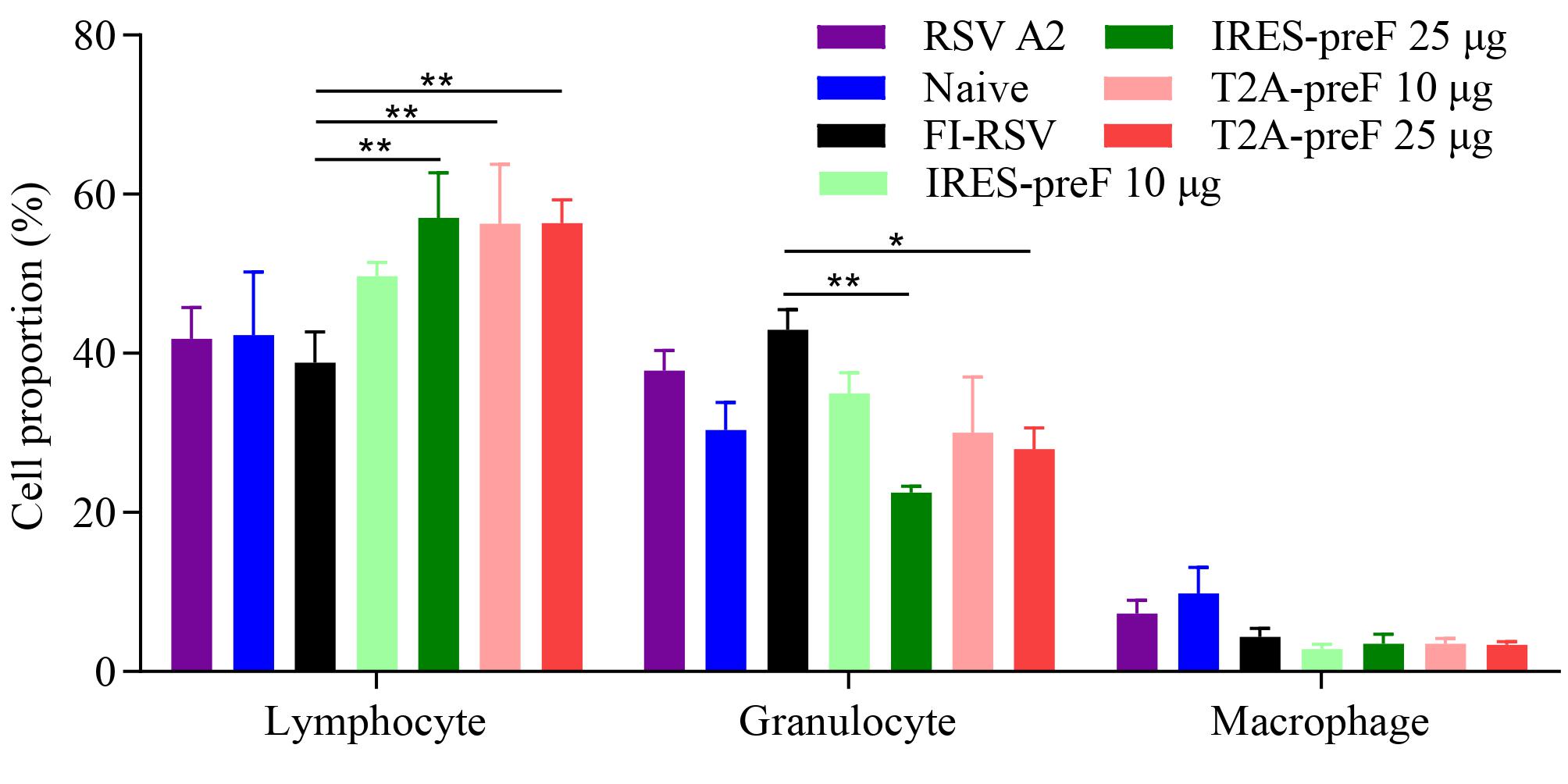

Supplement: Supplementary Figure 3 — The proportion of lymphocytes, granulocytes and macrophages in the BALF was analyzed by flow cytometry (n = 5). The data are presented as the mean ± SEM. All the data are representative of three independent experiments. A two-way ANOVA with Tukey’s multiple comparisons test was conducted, *p < 0.05; **p < 0.01. [file Image3.jpeg]
